# Improvement of surgical skills in students using a newly developed 3D printed osteotomy model of a partially retained wisdom tooth

**DOI:** 10.1186/s12909-025-08394-y

**Published:** 2025-12-10

**Authors:** Katharina Schaffrath, Mark Ooms, Anna Bock, Marie Sophie Katz, Frank Hölzle, Ali Modabber

**Affiliations:** Department of Oral and Maxillofacial Surgery, University Hospital RWTH, Pauwelsstraße 30, Aachen, 52074 Germany

**Keywords:** Dental student, Osteotomy, Model, Wisdom tooth, Oral surgery, 3-D print

## Abstract

**Objectives:**

Against the background of digitalization, practicing on 3-D models for dental education has become more important. To improve the surgical skills of dental students, this study aimed to develop a 3-D-printed model for osteotomy of a partially retained lower wisdom tooth and evaluate surgical skills of students with practical experience acquired through phantom exercises compared to students who acquired experience only by assisting oral surgeons.

**Materials and methods:**

We developed a 3-D model that allows dental students to perform an osteotomy of a partially retained real wisdom tooth in region 48. The model was evaluated by oral surgeons (OS; *n* = 5), students with phantom experience (PE; *n* = 26), and students with experience with clinical assistance (CE; *n* = 29). Additionally, student performance was rated.

**Results:**

The OS, PE, and CE groups all evaluated the model as suitable for student courses, except for the gingiva mask. The CE group developed slightly better. In the exercise, the PE group showed better incision results, while the CE group was slightly better in the preparation of the working field.

**Conclusions:**

The osteotomy model is suitable for hands-on courses for dental students, but the gingiva should be improved. However, phantom exercise cannot replace clinical experience.

**Clinical relevance:**

To improve education, knowledge and self confidence of students in dental school.

**Supplementary Information:**

The online version contains supplementary material available at 10.1186/s12909-025-08394-y.

## Introduction

 Evidenced by the rising number of publications, digitalization in dentistry is developing rapidly [1]. More and more practices and clinics own 3-D scanners and printers, as they are used in chairside treatment [2] as well as for operative guides in fully guided implantation [3]. Likewise, dental education has drastically changed in recent years. Especially during the COVID-19 pandemic, teaching formats became more digital. Although students tolerate high levels of digital learning and benefit from online courses according to theoretical lectures [4], dentistry in particular cannot be taught without practical experience. While improved learning applications increase motivation, surgical skills themselves can only be optimized through practice and repetition [5]. There is a big difference between the learning process in undergraduate studies and the combination of practical skills and responsibility that a qualified dentist has when treating patients. Practical training is therefore essential for dental students. In addition to the improvement of their manual skills, students gain an understanding of anatomical structures better by practicing in hands-on-courses [6].

Even though industrial models (Frasaco GmbH, Tettnang, Germany) and animal models, such as porcine jaws, continue to be used for courses [7], the number of 3-D-printed models is rising. They are used for interactive visual demonstration and operative planning [8] as well as for oral and maxillofacial surgeons who benefit from practicing submental flaps [9] and dysgnathia surgery [10] on phantom models. Antunes et al. developed a 3-D-printed oral surgery flap training model for undergraduates, which is promising for participants as it is realistic and useful for hands-on-training [11].

3-D models can be used to allow students to experience caries simulations [12, 13] and complete endodontic requirements such as practicing on the apical region [14], special ledge management [15], and even electronically determining working length [16]. All of these models have received good evaluations from students and participants. The skills for tooth preservation and smaller prosthodontics can be improved using the typodont model [17]. Even pediatric dentistry (pulpotomy and stainless steel pediatric crowns) can be practiced [18], which is quite meaningful considering that pediatric courses are rare.

In dental school, oral surgery seems to be underrepresented compared to other subjects, such as prosthodontics, in terms of teaching hours. However, practical phantom learning using 3-D-printed models to practice special oral surgery skills seems to be beneficial for students, teachers, and patients because students might have much more self-confidence and knowledge on their first operation with a real patient. Therefore, the aims of this study were to evaluate a realistic model for osteotomy that can be used and individually changed by every student, depending on their skill level, and to compare students’ surgical skills at different levels of education using this 3-D-printed model for the removal of a lower wisdom tooth.

## Materials and methods

### Preparation of the osteotomy model

**Fig. 1:**
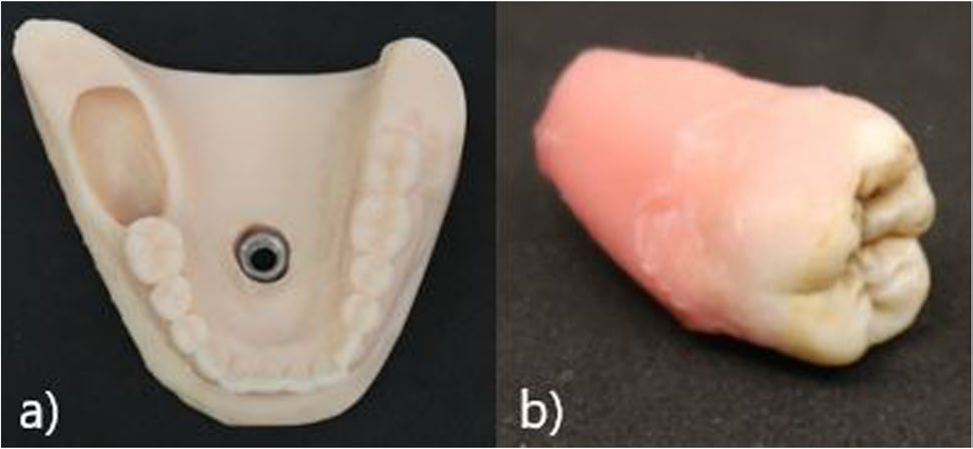
**a** 3D printed model base. **b** Wisdom tooth covered with wax

We designed a prototype of the model using a fully toothed plaster model of the lower jaw. To simulate the ascending course of the jaw, we extended the model with silicone in this region. A hole in the region of teeth 47 to 48 was milled to allow a tooth to be replaced later. Additionally, we milled a hole in the middle of the model to put in a fixation. 3-D scanning was performed using a T710 scanner (MEDIT), which allowed the digital model to be printed as often as needed. 3-D printing was carried out using a formlabs 3 printer with stereolithography (Photopolymer Resin SLA, model V3, formlabs, Somerville, Massachusetts, USA). One model was printed for each student. A nut was set into the bottom of the model with pattern resin (PATTERN RESIN LS, GC Germany) for fixing into a demo patient (Fig. 1a).

**Fig. 2:**
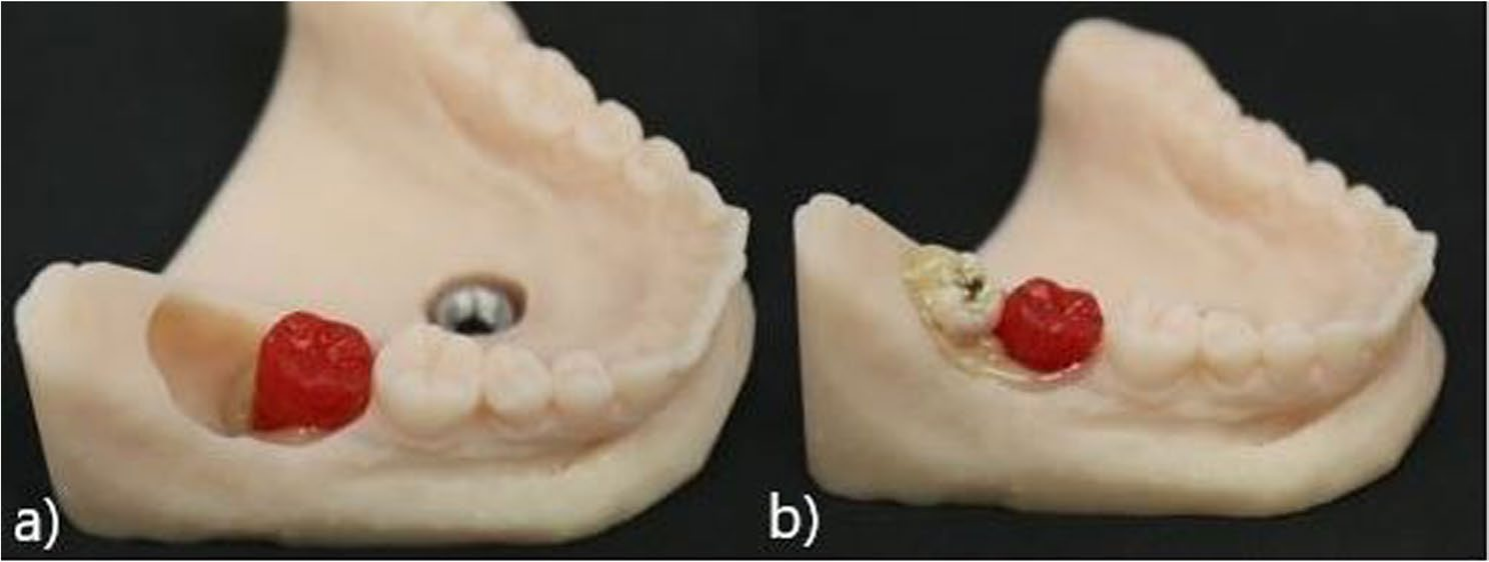
**a** Placed neighboured tooth. **b** Inserted wisdom tooth

To evaluate the status of the neighboring tooth, we designed a model of tooth 47 using modeling plastics (PATTERN RESIN LS, GC Germany) in red. We collected original wisdom teeth from patients who provided their teeth for a student training session. For this study, we used teeth with uncomplicated root designs, such as taproots. The disinfected teeth were submerged into pink modeling wax (Gebdi) only on the root side to simulate the periodontal gap (Fig. 1b). The crown remained clean. To simulate different levels of difficulty, the thickness of the wax can be changed. One model tooth 47 and one original wisdom tooth were positioned in a single model and fixed with Luxatemp (Luxatemp Star, DMG) simulating the bone (Fig. 2a). According to the level of difficulty, more or less of the surrounding bone can be placed. For this study, we designed a bone lamella from the distal side lying half over the occlusal surface of tooth 48 (Fig. 2b).

**Fig. 3:**
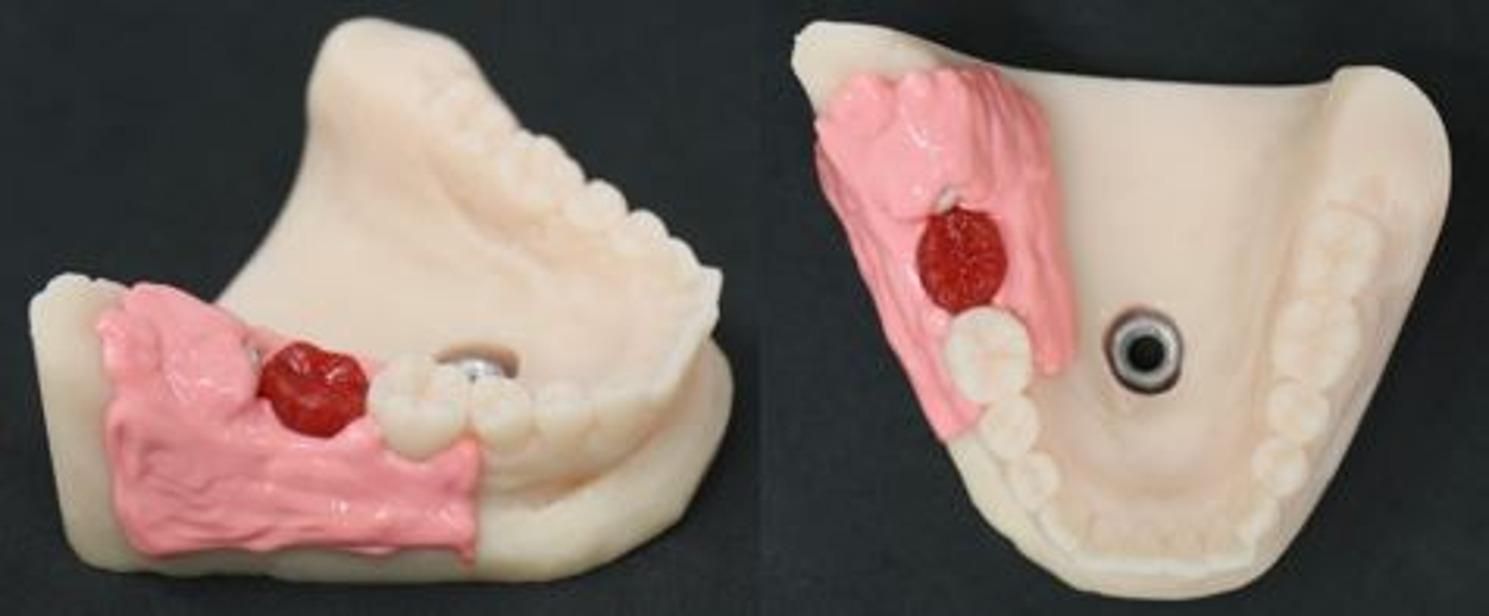
Finished model from buccal and occlusal

The same angulation was chosen for every model, with the crown lying at an angle of approximately 45° toward tooth 47. The depth depended on the size of the wisdom tooth but always enabled osteotomy, most likely class IIA or IIB, following the classification of Pell and Gregory [19]. To simulate the incision, uncovering, and suture, we used a gingiva mask (millable gum mask silicone, BRIEGELDENTAL) and attached it in a way simulating a partially retained tooth (Fig. 3).

### Evaluation of the model by oral surgeons

Before using the model for students, it was evaluated by five oral surgeons and dentists (OS; *n* = 5) with at least three years of surgical and teaching experience. All participants answered a questionnaire focusing on structure, realistic feeling, and exercise quality using a 10-point Likert scale.

### Osteotomy exercise by students

**Fig. 4:**
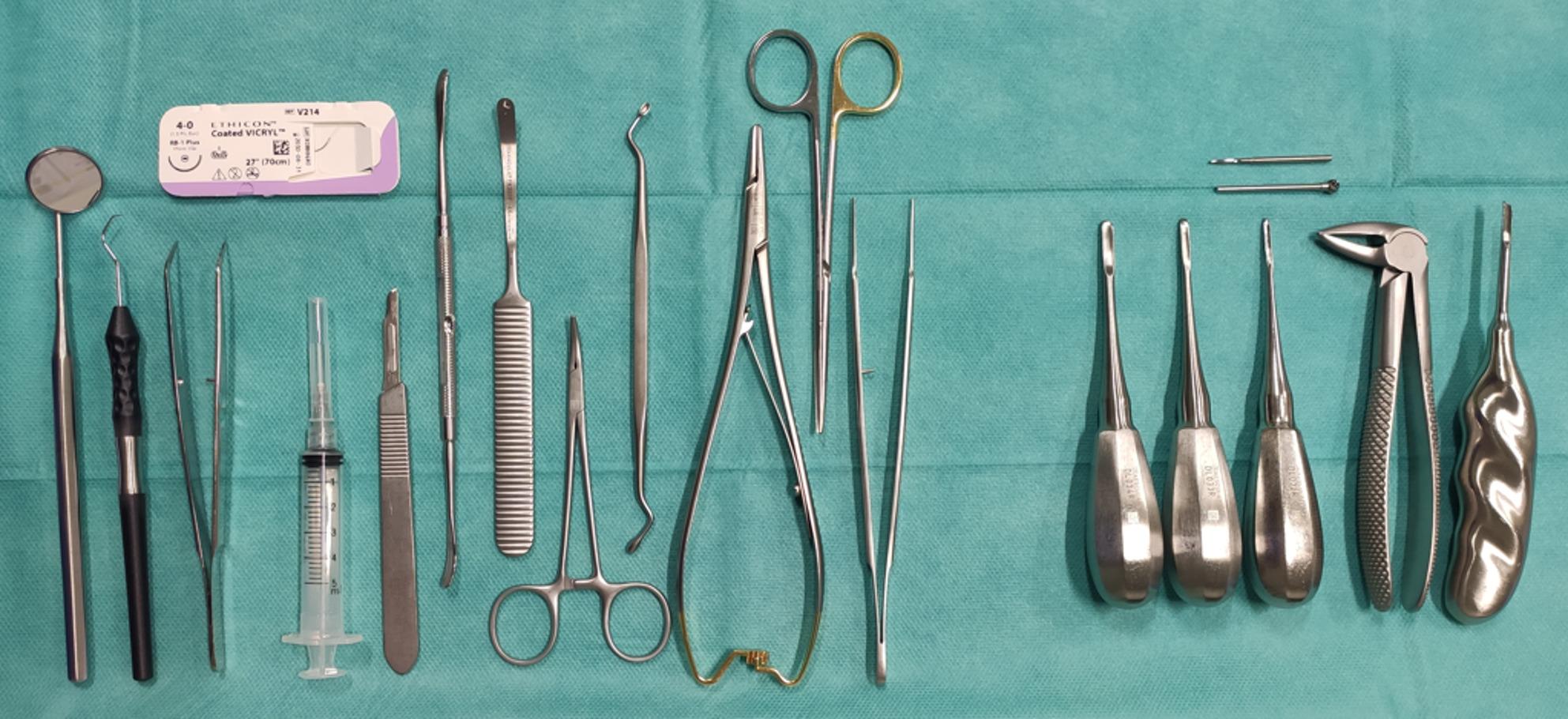
Requested preparation of the working field

Following the approval of the ethics committee of RWTH Aachen University (EK 24–370), which did not have any objections regarding data protection or the procedure, the exercise was performed. Informed consent to participate was obtained from all the participants. We compared the level of education of third-year undergraduate students with practical phantom exercise experience for one semester participating in a surgical course but no clinical experience (Group 1: PE; *n* = 37) to fifth-year undergraduate students who acquired their experience only by assisting oral surgeons, but without phantom exercise experience (Group 2: CE; *n* = 29). None of the students used the described model before. All participating students had theoretical knowledge of wisdom teeth removal from theoretical lectures. During the exercise, the following criteria were evaluated by a dentist on a scale divided into 0 (not fulfilled), 1 (uncertain), 2 (partially fulfilled), and 3 (fulfilled): preparation of working field (Fig. 4), demonstration of incision, performing anesthesia, and operation including suture. The maximum number of points was 57. All students answered an evaluation sheet (Supp. 1) after the osteotomy concerning structure, realistic feeling, and the quality of the exercise, followed by a personal evaluation (Supp. 2) focusing on their own skills using a 10-point Likert scale. Both of the evaluation questionnaires were developed for this study.

Thirty-seven third-year students in the PE group completed the exercise as part of their preclinical exam. Participation of the CE group was voluntary. All participants who missed more than eight questions (*n* = 5) or baseline information (*n* = 1) in the evaluation sheet or indicated that they had clinical experience besides the phantom exercise (*n* = 6) were excluded (*n* = 11). In total, 26 students were included in the PE group. In the CE group, all 29 fifth-year participants were included. For all students, this specific osteotomy model was new and unknown.

### Development of the evaluation sheets

All evaluation sheets and questionnaires were designed by two dentists of the Clinic for oral and maxillofacial surgery, hospital of RWTH Aachen as part of the dental preliminary medical examination.

### Statistical analysis

Data were expressed as numbers (with percentages) for categorial data and as medians (with interquartile range) for metric data. Testing for differences between groups in baseline data was performed using a chi-squared test, the Fisher–Freeman–Halton test was used for categorial data, and the Mann–Whitney test was used for metric data. Testing for differences between groups in evaluation data was performed using the Mann–Whitney test. Testing for correlation was performed by calculating Spearman correlation coefficients. P-values below 0.05 were considered significant. Statistical analysis was carried out using Excel 2016 (Microsoft, Redmond, USA), GraphPad Prism 10 (GraphPad Software, Boston, USA), and SPSS Version 28 (IBM, New York, USA).

## Results

### Evaluation of the osteotomy model by oral surgeons

Oral surgeons evaluated the gingiva, neighbored tooth, osteotomy tooth, bone, and fixation in terms of structure and realistic feeling. The best results were obtained for teeth and bone, while the worst results were obtained for the gingiva (Table 1).

**Table 1:** Evaluation of the model structure and realistic feeling by oral surgeons

Model	Structure	Realistic feeling
Gingiva	3 (5)	3 (5)
Neighboured tooth	9 (1)	9 (2)
Osteotomy tooth	9 (2)	10 (1)
Bone	7 (7)	8 (7)
Fixation	10 (9)	-

Data described as median (interquartile range) concerning model structure and realistic feeling for each question separately

They also evaluated the quality of exercise, finding that it was most suitable for students but was also suitable for residents (Table 2). Nevertheless, a combination of model exercises and clinical experience is valued as the best for preparing participants for patients’ treatment (10 (5)). Phantom models alone cannot replace clinical experience (3 (6)). For oral surgeons, model osteotomy enables realistic operation simulations in almost every rating point, except for the apical granuloma and incision with medium rating (Table 2).

**Table 2:** Evaluation of the quality of exercise by oral surgeons

Quality of exercise	OS
Suitable for residents	6 (6)
Suitable for students	10 (3)
Enables all operative steps	8 (6)
Simple and intuitive	9 (3)
Model replaces clinic experience	3 (6)
Combination of model and clinic is best	10 (5)
Only clinical experience eduacates	6 (6)
Model prepares for clinical treatment	7 (2)
Operation feels realistic	
Coloured neighboured tooth gives safety	6 (5)
Ascending jaw	10 (6)
Incision	6 (7)
Uncovering of the tooth	7 (5)
Periodontal gap	8 (6)
Relaxation of the tooth	10 (5)
Removal of the tooth	9 (6)
Apical granuloma	3 (6)

Data described as median (interquartile range) for each question separately

### Osteotomy exercise and evaluation by students

Concerning baseline information, there was a significant difference between the groups in age (PE: median = 22 (2), CE: median: 24 (7); *p* = 0.001) but not in sex (PE: m = 10, f = 16; CE: m = 7, f = 22, *p* = 0.381). During the analysis of the exercise results, the CE group achieved better results in preparing their working field in terms of mirror, probe, and tweezers (PE: 3 (0), CE: 3 (1); *p* = 0.033) and pliers and clamps (PE: 2 (2), CE: 3 (0); *p* = 0.001), while the PE group had better results in terms of respiratory (PE: 3 (1), CE: 3 (0); *p* = 0.015) (Table 3). Nevertheless, both groups showed good performance in preparing their working field. Regarding the incision, the PE group showed significantly better results, especially in drawing the marginal course (PE: 3 (0), CE: 3 (1); *p* = 0.006) and mesial discharge (PE: 3 (0), CE: 2 (2); *p* = 0.001) (Table 3). Both groups were able to save the lingual nerve (PE: 3 (0), CE: 3 (1); *p* = 0.613). Concerning other clinical steps like performing anesthesia (PE: 3 (1), CE: 3 (0); *p* = 0.008), uncovering of the tooth (PE: 3 (1), CE: 3 (0); *p* = 0.002), and removal of the tooth (PE: 3 (0), CE: 3 (0); *p* = 0.007) CE group performed better (Table 3), while values of the curettage of the apical granuloma were higher in the PE group (PE: 3 (0), CE: 3 (1); *p* = 0.026). However, the total score was equal between the groups (PE: 52 (4), CE: 54 (5); *p* = 0.131) (Table 3).

**Table 3:** Students exercise results

**Students exercise results**	**PE**	**CE**	**p-value**
Preparation working field			
Mirror, probe, tweezers	3 (0)	3 (1)	0,033
Syringe	3 (0)	3 (0)	1,000
Scalpel	3 (0)	3 (0)	1,000
Rasparatory	3 (1)	3 (0)	0,015
Drill	3 (0)	3 (0)	0,291
Lever	3 (0)	3 (0)	0,132
Pliers and clamps	2 (2)	3 (0)	0,001
Sharp spoon	3 (0)	3 (0)	0,291
Needle holder and stitch	3 (0)	3 (0)	0,132
Demonstration of Incision			
Drawing marginal incision	3 (0)	3 (1)	0,006
Protection of the lingual nerve (buccal direction)	3 (0)	3 (1)	0,613
Mesial discharge	3 (0)	2 (2)	0,001
Operation			
Performing anesthesia	3 (1)	3 (0)	0,008
Preparation of operation field	3 (0)	3 (0)	0,062
Uncovering the tooth	3 (1)	3 (0)	0,002
Removal of the tooth	3 (0)	3 (0)	0,007
Sound neighboring tooth	3 (0)	3 (0)	1,000
Curettage of apical granuloma	3 (0)	3 (1)	0,026
Suture	3 (0)	3 (0)	0,493
Total score	52 (4)	54 (5)	0,131

Data described as median (with interquartile range) for each question separately for groups (PE vs. CE); p-value corresponding to testing for differences between groups with Mann Whitney test

*abbreviations*: *PE* Students with practical experience on phantom exercise, *CE* Students without practical experience, experience acquired by assisting oral surgeons

In general, the model was evaluated with good results but was evaluated better by the CE group in terms of model structure (PE: 7.5 (3.3), CE: 10 (2); *p* = 0.001). All students indicated that the gingiva mask was not adequate (Table 4) because it often tears.

**Table 4:** Evaluation of the model by students

Evaluation Model structure	PE	CE	*p*-value
Gingiva	3 (3)	2 (5)	0,704
Neighboured tooth	8 (3)	10 (2)	0,030
Osteotomy tooth	10 (2)	10 (0)	0,002
Bone	9 (2)	8,5 (2)	0,318
Fixation	3 (6)	10 (3)	0,001
*Total (model structure)*	*7*,*5 (3*,*3)*	*10 (2)*	*0*,*001*
Realistic feeling			
Gingiva	4 (4)	4 (6)	0,844
Neighboured tooth	8 (4)	8 (4)	0,227
Osteotomy tooth	10 (1)	10 (0)	0,019
Bone	8 (3)	8 (3)	0,311
*Total (realistic feeling)*	*7*,*5 (3)*	*8 (3)*	*0*,*324*
By perceiving I feel prepared for clinical practice:			
Ascending jaw	5 (4)	6 (3)	0,233
Periodontal gap	5,5 (4)	5 (5)	0,425
Relaxation of the tooth	7 (3)	8 (3)	0,811
Apical granuloma	5 (5)	7 (3)	0,062
Incision	7,5 (6)	8 (2)	0,377
Uncovering of the tooth	7,5 (3)	9 (2)	0,050
Coloured neighboured tooth gives safety	8 (4)	8 (3)	0,437
*Total (preparation for clinic)*	*7 (2*,*5)*	*8 (3)*	*0*,*305*

Data described as median (with interquartile range) for each question separately for groups (PE vs. CE); p-value corresponding to testing for differences between groups with Mann Whitney test

*abbreviations*: *PE* Students with practical experience on phantom exercise, *CE* Students without practical experience, experience acquired by assisting oral surgeons; Total build as a median of its section

In general, all students thought that the model was suitable for increasing the experience of students and residents. All participants (PE: 8.5 (3), CE: 10 (1) and OS: 10 (5)) agreed that the combination of practicing on the model with clinical experience is the best option to prepare for daily practice (Table 5).

**Table 5:** What students think, the model is suitable for

Evaluation by students What I think about the model…	PE	CE	*p*-value
Suitable for residents	6,5 (3)	9 (4)	0,001
Suitable for students	7,5 (2)	10 (1)	0,001
Model replaces clinic experience	2,5 (3)	3 (4)	0,931
Combination of the model and clinic is best	8,5 (3)	10 (1)	0,017
Only clinical experience eduacates	6 (5)	5 (5)	0,377
Enables all operative steps	4,5 (3)	6 (4)	0,038
Simple and intuitive	7,5 (2)	8 (3)	0,069
Level of difficulty fits to my level of knowledge	9 (3)	9 (2)	0,186
Model prepares for clinical treatment	7 (3)	8 (3)	0,140
Skills can be improved by the model	8 (2)	9 (2)	0,021

Data described as median (with interquartile range) for each question separately for groups (PE vs. CE); p-value corresponding to testing for differences between groups with Mann Whitney test

*abbreviations*: *PE* Students with practical experience on phantom exercise, *CE* students without practical experience, experience acquired by assisting oral surgeons

To avoid any falsification of the results, we excluded a correlation between the exercise results (total score) and the total evaluation values of model structure (PE: ɸ = 0.280 (0.165), CE ɸ = 0.257 (0.179)), realistic feeling (PE: ɸ = 0.222 (0.275), CE: ɸ = 0.346 (0.066)), and preparation for clinic (PE: ɸ = 0.241 (0.237), CE: ɸ = 0.160 (0.407)).

## Discussion

The evaluation of the model was an important step in giving students the opportunity to practice and pass their preclinical exam. This exam was performed for the first time as there was a change in the systematic structure of dental studies in Germany. Against this background it was necessary for us as teaching dentists to find an opportunity to perform the exam. Therefore, we developed this model. This leads to one limitation of this study: the PE group did the exercise as a real exam, while the CE group participated voluntarily. To reduce bias, we chose evaluation sheets strictly and excluded those that were not fulfilled ordinary. Another limitation of this study is that the evaluation sheets were not validated. Moreover, not every model is exactly the same due to variations in the roots and sizes of the original wisdom teeth. Against this background, the level of difficulty can differ.

However, the students particularly liked that they had to remove a real tooth, as it makes the exercise more realistic. Thune et al. reported that the use of natural teeth provide better educational outcomes than plastic models [20]. Actually, it is an advantage of the model that it can be used individually, as it matches personal requirements, as shown by Arroyo-Bote et al. [17]. In future exercises, students should be able to choose the teeth, design the expression of the periodontal gap, or try different tooth positions according to skill level. Just as Reymus et al. concluded from their study, 3-D printing technology offers new possibilities for dental schools to create their own customized teaching models according to the specific curricula [21]. In our case, the base of the models can be recycled and can be filled with a new tooth as another advantage. Additionally, the simple and fast production and reproduction are important arguments for students who already pay a lot of money for materials and instruments [22].

Nevertheless, students and oral surgeons criticized the quality of the gingiva mask, as reported by Hanisch et al. [23]. This could be a hint to develop printable gingiva. Feng et al. reported that their gingiva mask is often describes as too fragile [24]. That is consistent with out oberservation. It was possible to perform sutures on the model, but the material used for the gingiva mask torn often. Students and oral surgeons evaluated it as not flexible enough. Factors that are also missing from the model are the periosteum and the inferior alveolar nerve. In future developments, these factors should be improved to provide a more realistic feeling. To complete the realistic experience, the possibility of X-rays, which is not possible yet, could be discussed. If future developments include nerves, the use of X-rays will be particularly important for diagnostic and positional relationships to the roots.

We found that students without phantom experience (only knowledge from theoretical lectures and experience assisting oral surgeons) chose significant other incision than those with phantom experience. Although they were able to save the lingual nerve through incision in a vestibular direction, they often did not extend it to tooth 47 or even tooth 46; instead, they put the discharge directly in region 48. Therefore, there remains the suspicion that there is not enough sight by assisting oral surgeons. Often, the working field is confusing due to saliva, blood, a moving tongue, and a reduced mouth opening, even for the surgeon. Because of these factors, teaching is even more difficult. Additionally, students are busy with suction and holding the tongue, so they cannot concentrate on the details of the operation. Although Peters et al. demonstrated that the number of practical exercises (in their case, surgical sutures) is a good determinant of competence improvement [25], Bock et al. showed that structured feedback has a major impact on improvement [26], which is simply not possible when students do not perform the operation themselves. Kulasegaram et al. stated that allowing learners to experiment before interacting with an instructor can improve their learning skills [27]. Against this background, it may be beneficial to allow students to discover their skills on a model before assisting oral surgeons in operations on real patients and to experience operating by themselves so they can benefit more while assisting.

Hattar et al. stated that the enhancement of students’ clinical skills and directed exposure is necessary to raise the level of perceived confidence, which will improve their current and future professional performance [28]. This is supported in our study by the assessment of oral surgeons and students at each level of experience, as they all agree that a combination of phantom and clinical experience is the best preparation for future daily practice as dentists. However, the evaluation showed that a model cannot replace clinical experience. Gaballah et al. found that one of the main reasons for a perceived lack of confidence is limited clinical exposure [29], while Karagkounaki et al. and De Christo et al. describes that 3D printed models can benefit preclinical education by creating different case scenarios that resemble real-life situations [30, 31].

Dobros et al. noted that the participants in the studies under review thoroughly recommend introducing 3-D models into hands-on practice [32]. This matches our recommendations, as students and oral surgeons gave positive feedback—Fifth-year students who haven’t had the opportunity to use phantom models before evaluated even better than third-year students.

## Conclusions

The osteotomy model is suitable for hands-on courses for dental students, but the gingiva mask must be improved in future developments. Fifth-year students without phantom experience would have liked to practice on this 3-D model, as it is now established in dental school. Although phantom exercise can improve surgical skills and confidence, it cannot replace clinical experience. Against this background we clearly recommend practicing on the model before the first clinical treatment.

## Supplementary Information

Supplementary Material 1.

Supplementary Material 2.

## Data Availability

The datasets used and analysed during the current study are available from the corresponding author on reasonable request. STL data to print the model base is available for free on request.

## Abbreviations

OS: Oral surgeons and dentists with at least three years of clinical experience who evaluated the osteotomy model
PE: Third-year dental students with practical experience through phantom exercises
CE: Fifth-year dental students without practical or phantom experience, experience acquired by assisting oral surgeons only
