# Supplementary material for: Improvement of surgical skills in students using a newly developed 3D printed osteotomy model of a partially retained wisdom tooth
Source: BMC Med Educ. 2025 Dec 10;26:67. doi: 10.1186/s12909-025-08394-y (PMC12801904; doi:10.1186/s12909-025-08394-y)
Supplement: Supplementary file 1 — Supplementary Material 1. [file 12909_2025_8394_MOESM1_ESM.docx]

**
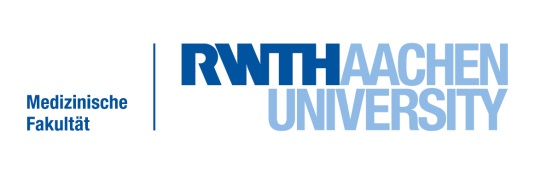
Study of Dentistry**

**1. Osteotomy of a wisdom tooth in the lower jaw**

|  | **Requirements according to the stage of study** | | |  |
| --- | --- | --- | --- | --- |
|  | **Not fulfilled** | **unsafe** | **Partially fulfilled** | **fulfilled** |
|  | **0** | **1** | **2** | **3** |
| Preparation of instruments |  |  |  |  |
| - Basic instruments | □  □  □  □  □  □  □  □  □ | □  □  □  □  □  □  □  □  □ | □  □  □  □  □  □  □  □  □ | □ |
| - Syringe and cannula |  |  |  | □ |
| - Scalpel |  |  |  | □ |
| - Rasparatorium according to Freer and Williger |  |  |  | □ |
| - Drill and burr |  |  |  | □ |
| - Bein lever and root elevator |  |  |  | □ |
| - Root remover forceps and clamps |  |  |  | □ |
| - Sharp spoon |  |  |  | □ |
| - Needle holder, suture, and thread scissors |  |  |  | □ |
| Demonstrating the incision   - Marking the incision (marginal region 7) - Incision: distobuccal (preserving the lingual nerve) - Incision: relief of the buccal region 7 or continuation to 6) |  |  |  |  |
|  | □  □  □ | □  □  □ | □  □  □ | □ |
|  |  |  |  | □ |
|  |  |  |  | □ |
| Performing the surgery |  |  |  |  |
| - Conductive anesthesia performed | □  □  □  □  □  □  □ | □  □  □  □  □  □  □ | □  □  □  □  □  □  □ | □ |
| - Incision and exposure of the surgical area |  |  |  | □ |
| - Exposure of the tooth with a drill (up to the cementoenamel junction) |  |  |  | □ |
| - If necessary, sectioning and removal of the tooth |  |  |  | □ |
| - Adjacent tooth intact |  |  |  | □ |
| - Curettage/cleaned alveolus - Suture |  |  |  | □ |
|  |  |  |  | □ |
|  |  |  |  |  |

| **points:** | _________/ 57 |
| --- | --- |
|  | |
