# Supplementary material for: Improvement of surgical skills in students using a newly developed 3D printed osteotomy model of a partially retained wisdom tooth
Source: BMC Med Educ. 2025 Dec 10;26:67. doi: 10.1186/s12909-025-08394-y (PMC12801904; doi:10.1186/s12909-025-08394-y)
Supplement: Supplementary file 2 — Supplementary Material 2. [file 12909_2025_8394_MOESM2_ESM.docx]

**Evaluation**

**Demonstration model for students**

**Osteotomy of a partially impacted tooth 48**

**General**

1.1. Gender: □ f □ m

1.2. Age: _____________

1.3. Semester: □ 6th semester □ 7th semester □ 8th semester

□ 9th semester □ 10th semester

1.4. Previous experience:

□ No experience with wisdom tooth osteotomies

□ Previously observed wisdom tooth osteotomy on a patient

□ Previously assisted with wisdom tooth osteotomy on a patient

□ Previously performed wisdom tooth osteotomy on a patient myself

□ Previously performed wisdom tooth osteotomy on a model myself

Model structure

2.1. I think the texture and structure of the model are good.

Gingiva

Fully applies ❑ ❑ ❑ ❑ ❑ ❑ ❑ ❑ ❑ ❑ not applicable at all

Adjacent tooth

Fully applies ❑ ❑ ❑ ❑ ❑ ❑ ❑ ❑ ❑ ❑ not applicable at all

Osteotomy tooth

Fully applies ❑ ❑ ❑ ❑ ❑ ❑ ❑ ❑ ❑ ❑ not applicable at all

Bone structure

Fully applies ❑ ❑ ❑ ❑ ❑ ❑ ❑ ❑ ❑ ❑ not applicable at all

Attaching the model to the phantom head

Fully applies ❑ ❑ ❑ ❑ ❑ ❑ ❑ ❑ ❑ ❑ not applicable at all

2.3 The feel of the model gives me a realistic training experience.

Gingiva

Fully applies ❑ ❑ ❑ ❑ ❑ ❑ ❑ ❑ ❑ ❑ not applicable at all

Adjacent tooth

Fully applies ❑ ❑ ❑ ❑ ❑ ❑ ❑ ❑ ❑ ❑ not applicable at all

Osteotomy tooth

Fully applies ❑ ❑ ❑ ❑ ❑ ❑ ❑ ❑ ❑ ❑ not applicable at all

Bone structure

Fully applies ❑ ❑ ❑ ❑ ❑ ❑ ❑ ❑ ❑ ❑ not applicable at all

Quality of exercises

3.0 I think the models is useful for clinicians/residents to practice on.

Fully applies ❑ ❑ ❑ ❑ ❑ ❑ ❑ ❑ ❑ ❑ not applicable at all

3.1 I think the models is useful for students to practice on.

Fully applies ❑ ❑ ❑ ❑ ❑ ❑ ❑ ❑ ❑ ❑ not applicable at all

3.2 I think clinical experience can be replaced by exercises on models.

Fully applies ❑ ❑ ❑ ❑ ❑ ❑ ❑ ❑ ❑ ❑ not applicable at all

3.3 I think a combination of model exercises and clinical experience is ideal for preparing for everyday practice.

Fully applies ❑ ❑ ❑ ❑ ❑ ❑ ❑ ❑ ❑ ❑ not applicable at all

3.4 I believe that only clinical experience can adequately prepare you for everyday practice.

Fully applies ❑ ❑ ❑ ❑ ❑ ❑ ❑ ❑ ❑ ❑ not applicable at all

3.5 The model allows all surgical steps to be performed and practiced.

Fully applies ❑ ❑ ❑ ❑ ❑ ❑ ❑ ❑ ❑ ❑ not applicable at all

3.6 I find the model easy and intuitive to use.

Fully applies ❑ ❑ ❑ ❑ ❑ ❑ ❑ ❑ ❑ ❑ not applicable at all

3.7 I find the level of difficulty of the exercise appropriate in relation to my level of knowledge.

Fully applies ❑ ❑ ❑ ❑ ❑ ❑ ❑ ❑ ❑ ❑ not applicable at all

3.8 I think that practicing on the model prepares students well for everyday working life.

Fully applies ❑ ❑ ❑ ❑ ❑ ❑ ❑ ❑ ❑ ❑ not applicable at all

3.9 I find that my own skills can improve through practicing with the model.

Fully applies ❑ ❑ ❑ ❑ ❑ ❑ ❑ ❑ ❑ ❑ not applicable at all

3.10 Before performing the exercise, I assess my ability to osteotomize a wisdom tooth as follows:

very good ❑ ❑ ❑ ❑ ❑ ❑ ❑ ❑ ❑ ❑ very bad

3.11 After performing the exercise, I assess my ability to osteotomize a wisdom tooth as follows:

very good ❑ ❑ ❑ ❑ ❑ ❑ ❑ ❑ ❑ ❑ very bad

Surgical procedure

4.1 By consciously perceiving the structure or the surgical procedure, I have experienced added value in my preparation for everyday clinical practice.

Ascending branch

Fully applies ❑ ❑ ❑ ❑ ❑ ❑ ❑ ❑ ❑ ❑ not applicable at all

Periodontal gap

Fully applies ❑ ❑ ❑ ❑ ❑ ❑ ❑ ❑ ❑ ❑ not applicable at all

Loosening of the tooth

Fully applies ❑ ❑ ❑ ❑ ❑ ❑ ❑ ❑ ❑ ❑ not applicable at all

Periapical granuloma

Fully applies ❑ ❑ ❑ ❑ ❑ ❑ ❑ ❑ ❑ ❑ not applicable at all

Cutting

Fully applies ❑ ❑ ❑ ❑ ❑ ❑ ❑ ❑ ❑ ❑ not applicable at all

Preparation and exposure of the tooth

Fully applies ❑ ❑ ❑ ❑ ❑ ❑ ❑ ❑ ❑ ❑ not applicable at all

Colored adjacent tooth

Fully applies ❑ ❑ ❑ ❑ ❑ ❑ ❑ ❑ ❑ ❑ not applicable at all

| 5. What did I particularly like? |
| --- |
| 6. What can be improved? |
